# Endophytic extract Zhinengcong alleviates heat stress-induced reproductive defect in *Solanum lycopersicum*

**DOI:** 10.3389/fpls.2022.977881

**Published:** 2022-08-25

**Authors:** Xiaoshuang Cui, Shangjia Liu, Lina Zhang, Xinping Guo, Ting Li, Xiaoyu Zhang, Qingbin Wang, Weiqing Zeng, Jiabao Huang, Qiaohong Duan, Yunyun Cao

**Affiliations:** ^1^State Key Laboratory of Crop Biology, Shandong Agricultural University, Tai’an, Shandong, China; ^2^College of Horticulture Science and Engineering, Shandong Agricultural University, Tai’an, Shandong, China; ^3^Shandong Pengbo Biotechnology Co., Ltd., Tai’an, China; ^4^Trait Discovery, Corteva Agriscience, Johnston, IA, United States

**Keywords:** endophytic fungus extract, heat stress, pollen development, pollen viability, reactive oxygen species, *Solanum lycopersicum*

## Abstract

High temperature negatively affects reproductive process significantly, leading to tremendous losses in crop quality and yield. Zhinengcong (ZNC), a crude extract from the endophytic fungus *Paecilomyces variotii*, has been shown to improve plant growth and resistance to biotic and abiotic stresses. We show here that ZNC can also alleviate heat stress-induced reproductive defects in *Solanum lycopersicum*, such as short-term heat-induced inhibition on pollen viability, germination and tube growth, and long-term heat stress-induced pollen developmental defects. We further demonstrated that ZNC alleviates heat stress by downregulating the expressions of ROS production-related genes, RBOHs, and upregulating antioxidant related genes and the activities of the corresponding enzymes, thus preventing the over accumulation of heat-induced reactive oxygen species (ROS) in anther, pollen grain and pollen tube. Furthermore, spraying application of ZNC onto tomato plants under long-term heat stress promotes fruit and seed bearing in the field. In summary, plant endophytic fungus extract ZNC promotes the reproductive process and yield of tomato plants under heat stress and presents excellent potential in agricultural applications.

## Introduction

Due to global warming, high temperature stress has gradually become a serious challenge for crop production. Heat stress causes damages on plants during both vegetative and reproductive developments. Fruit and seeds, major edible organs of many crops, are mainly derived from sexual reproduction. However, the reproductive process as well as the development of floral organs, particularly pollen (Muller and Rieu, 2016; Smith, 2019), are very sensitive to high temperature (Hoshikawa et al., 2021). The duration of high temperature can be short-term or long-term. Short-term high temperature at anthesis compromises pollen viability, germination and tube elongation (Muhlemann et al., 2018). Long-term high temperature during pollen development results in severe defect on pollen development as well as pollen growth, compromising the seed set (Muhlemann et al., 2018). Both short-term and long-term heat stress can result in substantial loss in fruit quality and yield. Understanding how high temperature affects pollen development and pollen tube growth, and finding effective and economic ways to alleviate heat stress, will greatly promote the yield of many crops.

Tomato (*Solanum lycopersicum*), a worldwide cultivated vegetable crop, grows optimally between 20°C and 26°C and is able to endure extreme temperatures above 15°C and below 33°C (Pan et al., 2021). However, in tomato production, especially in greenhouses or protected facilities, temperature often can rise above 35°C, even to 40°C. In general, the ambient temperature becomes a heat stress when it is 10–15°C higher than the optimum range (Hoshikawa et al., 2021). Heat is the major abiotic stress affecting tomato fruit set. Previous study pointed out that the decreased fruit set of tomato is closely related to lower pollen viability under heat condition (Firon et al., 2006). Moreover, most commercial tomatoes are not tolerant to heat, and thermotolerance trait in tomato is hard to improve due to its low heritability (Hazra et al., 2009).

Zhinengcong (ZNC), a crude extract from the endophytic fungus *Paecilomyces variotii*, is able to improve plant growth and resistance to biotic and abiotic stresses. ZNC was found to promote the growth and immunity of *Arabidopsis thaliana* (Lu et al., 2019), potato yield, quality and resistance to *Phytophthora infestans* indoors and in the field (Cao et al., 2021). ZNC was also found to enhance tobacco resistance to *potato X virus* by promoting RNA silencing and the SA pathway (Peng et al., 2020). Recently, guanine was identified as the active component of ZNC and functions as immunity inducer to enhance rice resistance against rice sheath blight (Wang et al., 2022). ZNC could also improve cold and salt resistance of rice (Wang et al., 2019, 2020). However, whether ZNC can improve plant heat tolerance is unclear.

For long-term heat stress, high temperature induces premature degeneration of the tapetal cell layer, inhibits pollen development by restricting the supply of nutrients, enzymes, and precursors from tapetum (Baron et al., 2012; Omidi et al., 2014; Qi et al., 2018). It is known that reactive oxygen species (ROS) are important factors regulating tapetal degeneration in Arabidopsis, rice and tomato (Hu et al., 2011; Luo et al., 2013; Xie et al., 2014; Yu et al., 2017). Mutant plants defective in ROS scavenging activities, such as *mt-a-4b* and *cox11*, over accumulate ROS in the tapetum and show premature tapetal degeneration and pollen abortion (Hu et al., 2011; Luo et al., 2013). On the other hand, mutations of *RBOH*s, genes encoding NADPH oxidases related to ROS production, delay tapetal cell programmed cell death (PCD) and cause male sterility (Xie et al., 2014). These studies indicate that ROS level might be precisely controlled during pollen development. ROS serve as critical signaling molecules during not only pollen development, but also pollen tube growth (Speranza et al., 2012; Kaya et al., 2014; Smirnova et al., 2014; Xie et al., 2014). High temperature induces the over accumulation of ROS in pollen tube, inhibits tube growth and damages its integrity (Muhlemann et al., 2018).

In addition to the ROS signaling pathway, there are other molecules play important roles during pollen development in response to heat stress. Recently, it is reported that Phytochrome-Interacting Factor 4 (PIF4) homolog in tomato, SlPIF4, regulates the anther and pollen thermosensory to heat stress. PIFs, a small subset of basic helix–loop–helix (bHLH) family transcription factors, act as central regulators in the integration of light and temperature signals to optimize plant growth and development (Leivar and Quail, 2011). Mutation in SlPIF4 reduces temperature sensitivity of the tapetum thus restores pollen development in mild high temperatures (32°C/28°C, day/night for 4 days; Pan et al., 2021).

Zhinengcong, has been shown to improve plant growth and stress resistance (Wang et al., 2020). Whether ZNC plays a role in the reproductive growth in tomato and its underlying mechanisms are poorly understood. We show here that ZNC alleviates heat stress-induced reproductive defect and promote fruit yield in tomato, presenting excellent potential in agricultural applications.

## Materials and methods

### Plant material and growth conditions

Tomato (*Solanum lycopersicum* L. cv. Micro-Tom) plants were grown in mixed soil (vegetative soil and vermiculite, 3:1) in growth chamber at 25/ 20°C (day/night) and 16/8 h (day/night) for 6 weeks. These 6-week-old plants were sprayed with 10–30 ng/ml ZNC every 3 days (plants sprayed with water were used as controls). After 1-week pretreatment, plants were transferred to 37°C/27°C (day/night) for 5 days or at 32°C/ 27°C (day/night) until fruits ripen with continuous ZNC spraying every 3 days. Each treatment had three replicates and each replicate contained 10 plants.

### Pollen germination and viability assays

Newly opened flowers were selected from plants that were pre-treated with 10–30 ng/ml ZNC for a week, and incubated at 37°C for 4 h after spraying with ZNC. For pollen germination rate and pollen tube length measurement, pollen grains from these flowers were germinated *in vitro* at normal temperature (25°C) in pollen germination medium [PGM; 24% PEG4000, 1.6 mm boric acid, 2% sucrose, 20 mm HEPES pH 6.0, 30 mm Ca(NO_3_)_2_, 100 μm MgSO_4_, 5 mm KCl] for 2 h. For pollen viability test, pollen grains from the above treated flowers were stained in 0.001% fluorescein diacetate (FDA) and 20 μm propidium iodide (PI) in the dark for 20 min in pollen viability solution [PVS, containing 290 μm sucrose, 1.27 mm Ca(NO_3_)_2_, 0.16 mm H_3_BO_3_, and 1 mm KNO_3_]. For pollen growth on stigma, stigmas under normal temperature were pollinated with heat treated pollen grains or heat-treated stigmas were pollinated with normal pollen grains. At 6 h after pollination, these stigmas were fixed in Carnoy’s solution (methanol: acetic acid = 3:1) and stained in 0.1% aniline blue according to a reported method (Duan et al., 2014). Images were captured by the Nikon Eclipse Ni with DS-Ri2 digital camera.

### ROS staining assay

For the detection of ROS in pollen after short-term heat stress, newly opened flowers were sprayed with 10–30 ng/ ml ZNC, then incubated at 37°C for 4 h. Pollen grains from these treated flowers were stained with 20 μm 2′,7′-dichlorodihydrofluorescein diacetate (H_2_DCFDA) for 20 min, and then washed 3 times in PVS solution. Pollen grains were cultured for 40 min in PGM and pollen tubes were stained with 2 mg/ml nitroblue tetrazolium (NBT) for 8 min (Kou et al., 2021). For the detection of ROS in anther after long-term heat stress, flower buds from plants grown at 37°C/27°C (day/night) were sprayed with 20 ng/ ml ZNC every three days (the plants were pretreated with ZNC for 1 week before heat-stressed). After 5 days, the anthers were then soaked in sodium citrate solution (10 mm, pH 6.0) with 6 mm NBT for 10 min in the dark, and then boiled in a mixture of ethanol and glycerin (3,1) for 15 min. Images were captured by the Nikon Eclipse N*i* with DS-Ri2 digital camera. ROS intensity was quantified in ImageJ.

### Activities assays of antioxidant enzymes

Pollen after short-term heat stress were gathered and homogenized in liquid nitrogen and then resuspended in 2 ml phosphate buffer (0.05 M, pH 7.8). After centrifugation at 12,000 *g* for 20 min at 4°C, the supernatant was used for activity assays for SOD, CAT and POD. SOD activity was detected by NBT photochemical reduction method and an activity unit was defined as the amount of enzyme required to inhibit 50% NBT reduction (Beauchamp and Fridovich, 1971). POD activity was detected by measuring the absorbance of 470 nm due to guaiacol oxidation (Cakmak and Marschner, 1992). CAT activity was detected by measuring hydrogen peroxide consumption at 240 nm (Jablonski and Anderson, 1981).

### RNA extraction and quantitative real-time PCR analysis

Newly opened flowers after 4 h heat treatment or flower buds at tetrad or uninucleate stage after 5d heat treatment were collected as samples for RNA extraction. Plant RNA Mini Kit (DP420, Tiangen) was used for RNA extraction and reverse transcription. The qRT-PCR reactions were performed using ChamQ SYBR qPCR Master Mix (Q711-03, Vazyme) in qTOWER3 qPCR machine (Analytikjena, Germany) with three replications. Actin (GenBank accession number: AB199316) was used as an internal reference gene. Primers used are listed in Supplementary Table 1.

### Statistical analysis

All bar graphs and box plots were generated in Prism. Dots denote individual data points, and n denotes the number of pollen grains, pollen tubes, pistils, plants, fruits or observation views (each observation view from camera contains over 100 pollen grains or tubes). All data pass normality test in SPSS (Kolmogorov–Smirnov test and Shapiro–Wilk test, *p* > 0.05). Asterisk on the top of data bar indicates significant difference (two-tailed *t*-test, ^*^p < 0.05, ^**^p < 0.01) compared with the data bar on the far left, while asterisk above the bracket represents comparison between the two data bars indicated. The symbol n.s. indicates no difference. All experiments had 3 biological replicates with similar results.

## Results

### Short-term heat stress destroys pollen integrity and inhibits pollen growth

**Figure 1:**
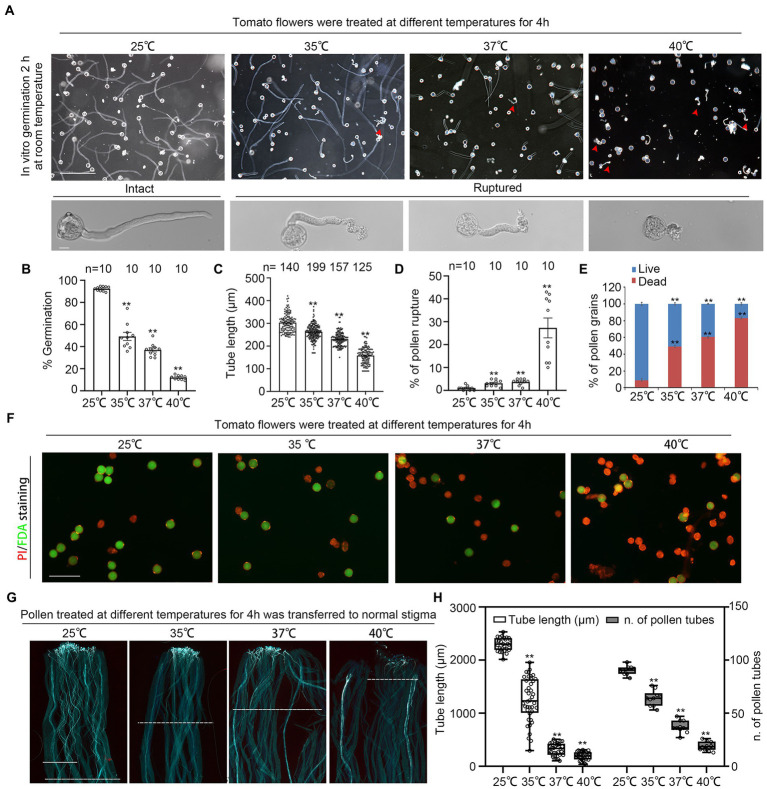
Short-term Heat stress decreases pollen viability and pollen growth. **(A)** Growth of pollen under 4 h treatment with different temperatures, 25, 35, 37, 40°C. Red arrows indicate ruptured pollen. Scale bars, 200 μm (upper panel), 10 μm (lower panel). **(B,C)** Quantification of pollen germination and tube length in **(A)**. n denotes the number of observation views **(B)** and pollen tubes **(C)**. Rupture of pollen in **(A)**. n denotes the number of observation views. **(E,F)** Dual-staining with fluorescein diacetate (FDA) and propidium iodide (PI) shows pollen viability under 4 h of treatment with different temperature (25, 35, 37, 40°C). Scale bars, 100 μm. **(G,H)** Growth of heat stress-treated pollen in normal pistils. Scale bars, 500 μm. Bars represent means ± SE m. Asterisks or n.s. right above the data bars indicate significant difference (two-tailed *t*-test, ^**^p < 0.01) or no significant difference compared with the data bars on the far left. All experiments have 3 biological replicates and they have similar results.

We firstly characterized the impact of high temperature on tomato reproductive process by treating newly opened flowers at 25°C, 35°C, 37°C or 40°C for 4 h, then germinated pollen from these treated flowers *in vitro* at normal temperature (25°C) for 2 h. The germination rate of pollen grains from 37°C and 40°C-treated flowers were only 38 and 12.07%, respectively, while the germination rate from flowers under normal temperature was 92% (Figures 1A, B). Pollen tube length was also significantly shorter under high temperature than that under normal temperature (Figures 1A,C). Furthermore, as high as 25.3% of the 40°C-treated pollen ruptured shortly after germination, while 35°C-and 37°C-treatment also increased pollen ruptures (although less than 5%) compared to 25°C, suggesting cell integrity damage under extreme high temperature (Figures 1A,D). Viability staining of pollen grains with fluorescein diacetate (FDA) and propidium iodide (PI) showed a drastic drop in pollen viability from 91.87% under 25°C to only 39.53 and 16.79%, respectively, under 37°C or 40°C treatment (Figures 1E,F). In addition, the growth of 37°C-and 40°C-treated pollen on normal stigmas were severely inhibited with the increasing of temperature (Figures 1G,H). However, normal pollen grew well on 37°C-and 40°C-treated stigmas (Supplementary Figure S1). Altogether, these results show that short-term high temperatures, 37°C and 40°C, had multiple inhibitory effect on pollen, such as decreasing pollen viability and pollen integrity, inhibiting pollen germination and pollen tube growth.

### ZNC alleviates short-term heat stress-caused inhibition on pollen growth

**Figure 2:**
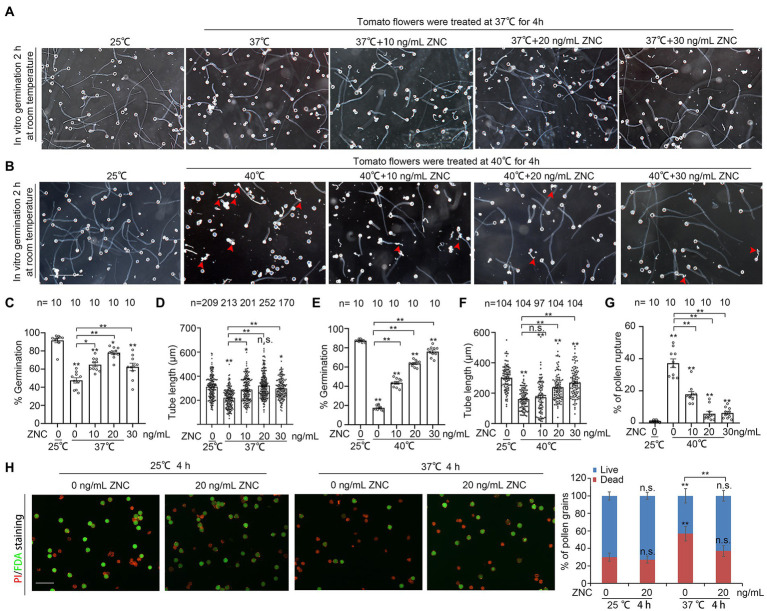
ZNC alleviates the decrease of pollen viability and pollen growth under short-term heat stress. **(A,B)** Effect of ZNC pretreatment on pollen growth under 4 h of 37°C or 40°C treatment. Scale bars, 200 μm. **(C,D)** Quantification of pollen germination **(C)**, tube length at 37°C **(D)** in **(A)**. n denotes the number of observation views **(C)** and pollen tubes **(D)**. **(E,G)** Quantification of pollen germination **(E)**, tube length **(F)** and pollen rupture at 40°C in **(G,B)**. Red arrows indicate ruptured pollen. n denotes the number of observation views **(E,G)** and pollen tubes **(F)**. Supplementary Figure S2 shows the effect of ZNC at different concentration under 25°C. **(H)** Dual-staining of FDA and PI shows the effect of ZNC pretreatment on pollen viability, under 4 h of 37°C heat stress. Scale bars, 100 μm in H. Bars represent means ± SE m. Asterisk on the top of data bar indicates significant difference (two-tailed *t*-test, ^*^p < 0.05, ^**^p < 0.01) compared with the data bar on the far left, while asterisk above the bracket represents comparison between the two data bars indicated. The symbol n.s. indicates no difference. All experiments have 3 biological replicates and they have similar results.

To examine whether ZNC has any beneficial effect on pollen under short-term heat stress, we pre-treated newly opened flowers with 10–30 ng/ml of ZNC, followed by 4 h of treatment under 37°C or 40°C, then germinated pollen from these treated flowers *in vitro* at 25°C. While the mock-treated flowers showed poor pollen germination of only 40% at 37°C, pollen from 20 ng/ml ZNC pre-treated flowers showed up to 78.10% germination rate under the same condition (Figures 2A,C). Pollen from 30 ng/ml ZNC pre-treated flower also showed similar germination rate under 40°C (Figures 2B,E). Similarly, the length of pollen tube from 20 ng/ml ZNC pre-treated flowers at 37°C and 30 ng/ml ZNC pre-treated flowers at 40°C is comparable to that from flowers at normal temperature (Figures 2A,B,D,F). The percentage of pollen rupture at 40°C was also greatly reduced in flowers with ZNC pretreatment (Figures 2B,G). As a control, we also tested the impact of ZNC treatment on pollen growth at normal temperature (25°C). We found the pollen germination at 25°C was not affected by ZNC at all, but pollen tube growth was promoted at low concentration (10 ng/ml) and inhibited at higher concentration (40 ng/ml; Supplementary Figures 2A,B). Altogether, these results suggest that ZNC pretreatment alleviates heat-induced pollen tube growth defect, pollen rupture as well as pollen viability defect.

### ZNC reduces heat-induced ROS accumulation in pollen and pollen tubes

**Figure 3:**
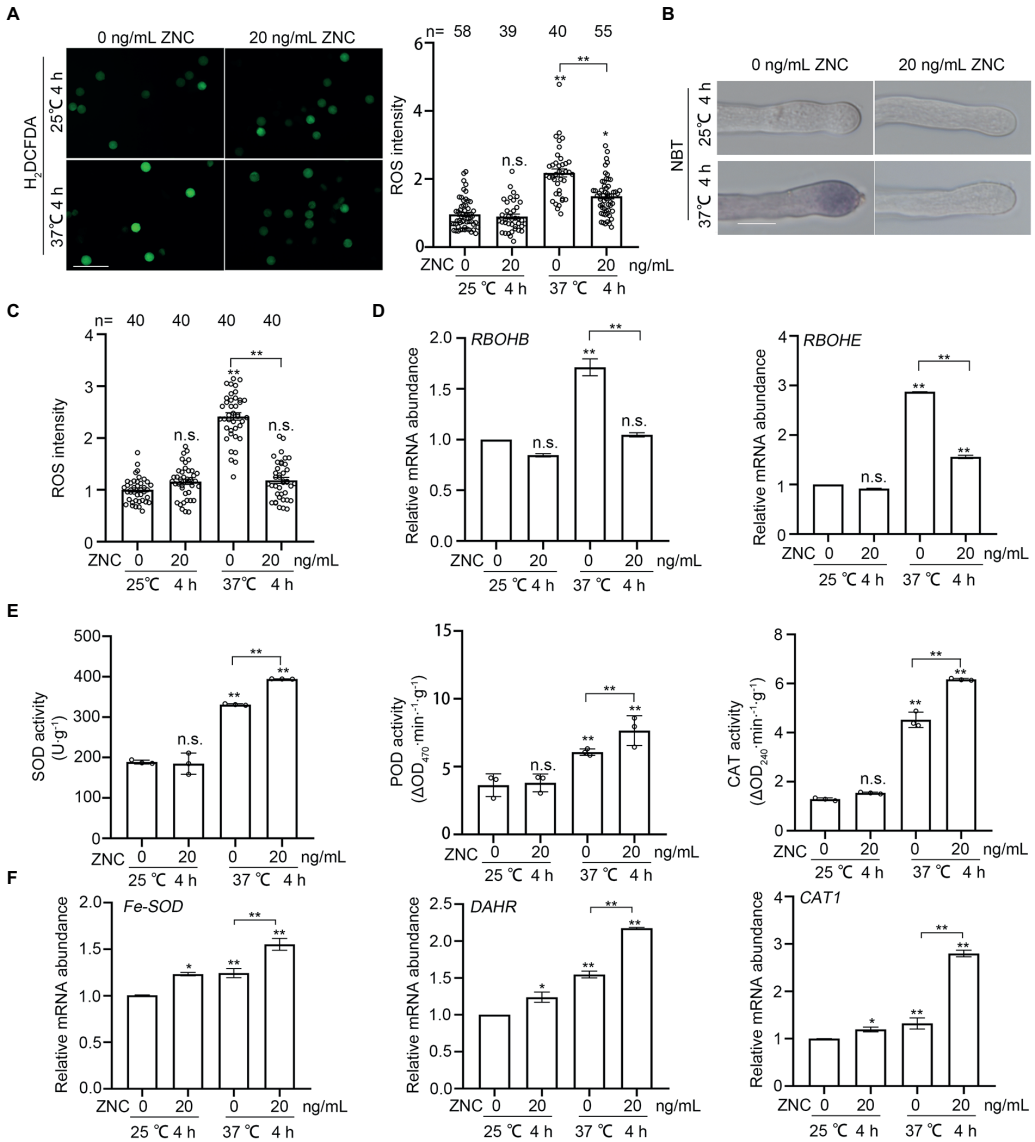
ZNC reduces short-term heat-induced ROS accumulation in pollen grains and pollen tubes. **(A)** H_2_DCFDA staining show ROS accumulation in pollen grains. Scale bar, 100 μm. ROS intensity was measured by ImageJ and ROS in pollen grains at 25°C without ZNC treatment were set at 1 for comparative analyses. n denotes the number of pollen grains. **(B,C)** NBT staining show ROS accumulation in pollen tubes. Scale bar, 10 μm. ROS intensity was measured by ImageJ and ROS in pollen tubes at 25°C without ZNC treatment were set at 1 for comparative analyses. n denotes the number of pollen tubes. **(D)** Expressions of *RBOH* genes, *RBOHB* and *RBOHE*, after 4 h at 37°C with ZNC treatment. **(E)** Expressions of anti-oxidant related genes, *Fe-SOD*, *DAHR* and *CAT1*, after 4 h at 37°C with ZNC treatment. **(F)** Enzyme activities of SOD, POD and CAT after 4 h at 37°C with ZNC treatment. Bars represent means ± SE m. Asterisk on the top of data bar indicates significant difference (two-tailed *t*-test, ^*^p < 0.05, ^**^p < 0.01) compared with the data bar on the far left, while asterisk above the bracket represents comparison between the two data bars indicated. The symbol n.s. indicates no difference. All experiments have 3 biological replicates and they have similar results.

High temperature induces the over accumulation of ROS in pollen tube, inhibits tube growth and damages its integrity (Muhlemann et al., 2018). To investigate whether ZNC alleviates heat-induced pollen defect by regulating ROS, we pre-treated newly opened flowers with 10–30 ng/ml ZNC or water-control, followed by 4 h of heat treatment at 37°C or 25°C, then stained the pollen grains from these treated flowers with H_2_DCFDA (2′,7’-Dichlorodihydrofluorescein diacetate). We found that ROS accumulation was induced by high temperature in pollen grains from flowers after 4 h treatment at 37°C (Figure 3A). ROS levels in ZNC-pretreated pollen grains at 37°C were slightly higher than that in pollen under normal temperature but significantly lower than that in mock-treated pollen at 37°C (Figure 3A). Nitroblue tetrazolium (NBT) staining of pollen tubes from these ZNC-and heat-treated flowers showed that ROS accumulation in the tip of pollen tubes was also increased by heat stress but reduced to normal level by ZNC pre-treatment (Figures 3B,C). These observations suggest that ZNC alleviates heat stress on pollen and pollen tubes by reducing ROS accumulation in pollen.

To investigate the role of ZNC in the regulation of plant ROS production and scavenging, we first examined the expression of *RBOH*s genes, *RBOHB* and *RBOHE*, which are highly expressed in mature anther and pollen (Yu et al., 2017). The expressions of two *RBOH* genes were both induced by heat, but reduced with ZNC treatment at 37°C (Figure 3D). Then, we examined the expressions of antioxidant-related genes, *Fe-SOD*, *DAHR*, and *CAT1* (Qi et al., 2018). The transcript levels of these three antioxidant-related genes increased after ZNC pretreatment under normal temperature (Figure 3E). They were also induced by high temperature and further induced by ZNC pretreatment (Figure 3E). Consistent with the changes in gene expression, the activities of superoxide dismutase (SOD), peroxidase (POD) and catalase (CAT) were significantly increased by 19, 26, and 36% in ZNC-pretreated compared with untreated anthers under high temperature, respectively (Figure 3F). Altogether, our results clearly demonstrated that ZNC pretreatment significantly reduced heat-induced ROS over-accumulation in tomato pollen, which are likely due to ZNC-triggered downregulation of *RBOH* genes and upregulation of antioxidant-related genes and their enzyme activities.

### ZNC rescues long-term heat-induced pollen developmental defect

**Figure 4:**
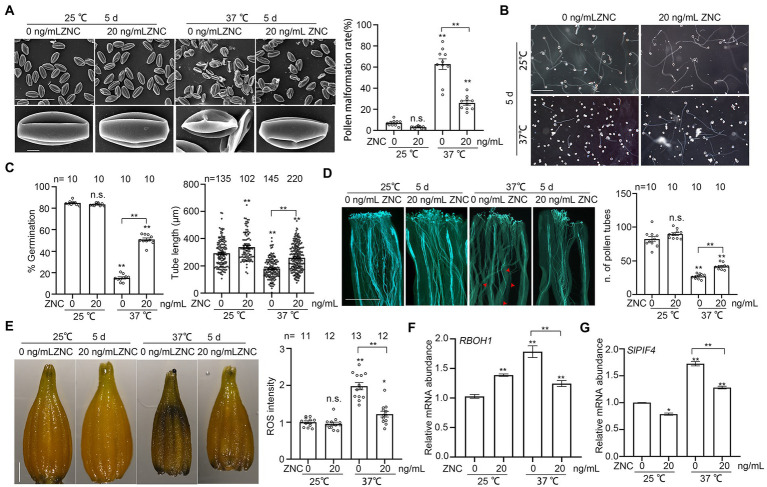
ZNC rescues long-term heat stress-induced pollen development defect. **(A)** Scanning electron microscope shows the morphology of pollen from plants with or without ZNC pretreatment, under 5 days of 25°C or 37°C treatment. Scale bar, 100 μm (upper panel), 5 μm (lower panel). **(B)** Growth of pollen tubes *in vitro* after 5 days of 25°C or 37°C treatment. Scale bar, 200 μm. **(C)** Quantification of pollen germination and tube length in **(B)**. n denotes the number of observation views or pollen tubes. **(D)** Growth of pollen tubes in unstressed pistils after 5 days of 37°C treatment. Red arrows indicate the positions that pollen tubes reached. Scale bar, 500 μm. n denotes the number of pistils. **(E)** NBT staining show the effect of ZNC pretreatment in alleviating ROS accumulation in anther. ROS intensity was measured by ImageJ and ROS in anthers at 25°C without ZNC treatment were set at 1 for comparative analyses. Scale bar, 1,000 μm in D. n denotes the number of anthers. **(F)** Expression of RBOH1 in anther after 5 d at 25°C or 37°C treatment. **(G)** Expression of SlPIF4 in anther after 5 d at 25°C or 37°C treatment. Bars represent means ± SE m. Asterisk on the top of data bar indicates significant difference (two-tailed *t*-test, ^*^p < 0.05, ^**^p < 0.01) compared with the data bar on the far left, while asterisk above the bracket represents comparison between the two data bars indicated. The symbol n.s. indicates no difference. All experiments have 3 biological replicates and they have similar results.

Long-term heat stress during floral organ development may result in pollen deformity or abortion (Pan et al., 2021). To examine the effect of ZNC on long-term heat stress induced pollen developmental defect, we pretreated tomato plants with 20 ng/ml ZNC followed by 5 days of 37°C treatment with continuous ZNC spray. Plants under 37°C-treatment produced more than 62% shriveled pollen grains, but treatment with 20 ng/ml ZNC reduced pollen abnormality to only 26% (Figure 4A). When germinated *in vitro* and *in vivo* on tomato pistils, pollen grains from these long-term heat-treated plants germinated poorly and tubes were significantly shorter than those from untreated control plants. However, both phenotypes were restored to normal by ZNC treatment (Figures 4B–D). Altogether, these results suggest that ZNC protects pollen growth under high temperature.

Since ZNC treatment significantly reduced the short-term heat-induced ROS over-accumulation in tomato pollen (Figures 3A,B), we tested the effect of ZNC on ROS accumulation in anthers from plants under long-term high temperature stress. NBT staining of anthers from plants under 5 days treatment at 37°C shows 1.98 times higher deposition of ROS than that of plants under normal temperature of 25°C (Figure 4E). However, ZNC treatment effectively alleviated the high temperature-induced ROS accumulation in anther (Figure 4E). We also examined the expression of *RBOH1*, which contributes to ROS production during tomato pollen development (Yan et al., 2020). The expression of *RBOH1* was upregulated around 2-times by heat, but down-regulated by ZNC treatment at 37°C (Figure 4F), suggesting that ZNC treatment reduces ROS production, which is induced by long-term heat stress during pollen development. These results suggest that ZNC is an effective modulator under high temperature by reducing the ROS accumulation.

Transcriptional factor SlPIF4 is important for extreme temperature tolerance by regulating temperature sensitivity of tapetum (Pan et al., 2021). We therefore examined whether the expression of *SlPIF4* is involved in ZNC’s ability to protect pollen development under high temperatures. Without the presence of ZNC, the expression of SlPIF4 was significantly upregulated in plants under high temperature as expected (Figure 4F). However, the presence of 20 ng/ml ZNC lowered the SlPIF4 expression to about 80% of the levels in no-ZNC-treated plants regardless if the plants were under normal or high temperature (Figure 4F). Altogether, these results suggest that ZNC protects pollen development under heat stress by reducing the thermosensory and reducing ROS over accumulation in anthers.

### ZNC raises fruit and seed bearing under long-term heat stress

**Figure 5:**
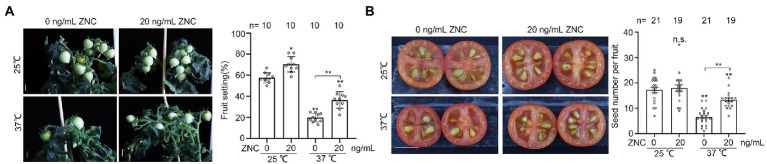
ZNC raises fruit and seed bearing under long-term high temperature stress. Plants of 6-week-old were placed under 32°C/27°C (day/night) until fruits are ripe with or without 20 ng/ml ZNC treatment. (% fruit setting = number of fruits / flowers ×100). **(A)** Fruit bearing from plants with or without ZNC pretreatment, under long-term treatment at 25°C or 37°C. **(B)** Seed set from plants with or without ZNC pretreatment, under long-term treatment at 25°C or 37°C. n indicates fruit number. Scale bars, 1 cm in **A**; 1 cm in **A**. n indicates the number of tomato plants. Bars represent means ± SE m. Asterisk on the top of data bar indicates significant difference (two-tailed *t*-test, ^*^p < 0.05, ^**^p < 0.01) compared with the data bar on the far left, while asterisk above the bracket represents comparison between the two data bars indicated. The symbol n.s. indicates no difference. All experiments have 3 biological replicates and they have similar results.

Since ZNC treatment improves pollen thermo-tolerance (Figures 1–4), we explored to what extent ZNC impact fruit setting under long-term heat stress. Tomato plants were grown under normal temperature for 6 weeks, then moved to 32°C/25°C (day/night) and grown until fruit ripen with or without continuous ZNC treatment. We found the fruit settings in tomato plants were higher with ZNC pretreatment at both 25°C and 37°C (Figure 5A). At 25°C, fruit setting increased from 57.7 to 70.4% with the presence of ZNC; while at 37°C, fruit setting increased from 20 to 40% with ZNC treatment. These observations suggest ZNC is able to promote tomato growth under both conditions. (Figure 5A). Meanwhile, seed number per fruit in 37°C-grown plants was increased from 6.57 to 13.32 with ZNC treatment, but showed no difference in 25°C-grown plants with or without ZNC treatment (Figure 5B). These results indicate that ZNC increases the efficiency of fruit bearing and seed formation under long-term heat stress.

## Discussion

High temperature greatly affects pollen development and pollen growth in plants, leading to tremendous loss in fruit quality and yield of many crops (Muller and Rieu, 2016; Smith, 2019; Hoshikawa et al., 2021). In this study, we showed that short-term heat stress-induced pollen growth defects and long-term heat stress-induced pollen developmental defects were both alleviated by ZNC pretreatment.

Under normal growth conditions, cellular ROS in plants remains balanced. However, high temperature stress induces excessive accumulation of ROS (Sheng et al., 2006). On one hand, long-term heat stress may cause over accumulation of ROS in the tapetum of the anther during pollen development, leading to premature degeneration of tapetum cells and therefore pollen developmental defect (Qi et al., 2018). It is likely that heat stress causes premature degeneration of tapetum as a consequence of high level of ROS, which results to a lack of nutrients in pollen during development, and ultimately leading to pollen abortion. On the other hand, short-term heat stress at anthesis and pollination causes overaccumulation of ROS in pollen grains and tips of pollen tubes, which impair pollen germination and tube growth, and compromise tube integrity (Muhlemann et al., 2018). Moreover, high level of ROS also cause oxidative damage to protein, DNA and lipid within plant cells (Qiu et al., 2019). Removal of ROS could improve pollen viability, germination and tube growth under heat. Antioxidant ascorbic acid and the NADPH oxidase inhibitor, DPI, rescue acute high temperature-induced reduction in pollen tube growth and integrity of tomato (Muhlemann et al., 2018). Melatonin, one of strong antioxidants, reduces ROS accumulation in tomato anthers and ultimately alleviates pollen abortion under high temperature (Qi et al., 2018). Therefore, the ability of plants in removing ROS is associated with its heat stress tolerance (Muhlemann et al., 2018). Plants have evolved an antioxidant system to scavenge ROS and reduce the oxidant damages caused by heat or other stresses, of which antioxidant enzymes (SOD, POD, and CAT) are vital parts (Yu et al., 2019). Our results show that ZNC reduces ROS accumulation not only in pollen grains and tubes under short-term heat stress, which enhances pollen tube growth and integrity (Figure 3), but also in anthers under long-term heat stress, which protect tapetum from early PCD and eventually ensure pollen proper development (Figure 4). These results suggest that the alleviation effect of ZNC on pollen is likely due to their abilities to reduce ROS generation and scavenging. In addition to ROS, a transcriptional factor SlPIF4-modulated pathway is another way regulating tapetal PCD (Pan et al., 2021). SlPIF4 directly interacts with the well-known tapetum development module DYT1-TDF1-AMS to regulate tapetal PCD. Knockout of SlPIF4 decreases thermosensitivity and enhances thermotolerance of anther. Our finding that SlPIF4 was upregulated by heat and then downregulated by ZNC (Figure 4F), suggests that ZNC enhances thermo-tolerance possibly by decreasing thermosensitivity of tapetum and repressing tapetal early PCD.

As a chemical fertilizer, ZNC promotes plant growth and defense against pathogens at a very low dosage (Yan et al., 2020). According to previous studies in the role of ZNC in stress resistance and growth, ZNC performs best in root growth of *Arabidopsis* at 1–100 ng/ml, bacterial resistance of *Arabidopsis* at 10–100 ng/ml, virus resistance of tobacco at 100–150 ng/ml, plants growth and oomycete resistance of potato at 1–100 ng/ml, which indicate that ZNC has high activity (Lu et al., 2019; Yan et al., 2020; Cao et al., 2021). Consistently, in our study, 20 ng/ml ZNC could effectively alleviate heat stress-induced inhibition on pollen grains and increase the production of tomato fruits and seeds (Figures 2, 4, 5). Due to its high activity, ZNC was used with extremely low dosage with very low cost, which shows great potential for commercial tomato farming. In summary, as an extract of endophytes, ZNC is environment friendly, and can be used as a new strategy to increase crop thermotolerance to improve summer crop production.

## Data availability statement

The original contributions presented in the study are included in the article/Supplementary material, further inquiries can be directed to the corresponding authors.

## Author contributions

QD conceptualized and designed the research plan and the experiments, interpretated the results, with contributions from JH and YC. XC and YC performed major experiments and data analysis with help from SL, XG, TL, LZ, and XZ. QD and YC led the writing process with help from JH, WZ, and QW. All authors contributed to the article and approved the submitted version.

## Funding

This work was supported in part by Research Start-up Fund from Shandong Agriculture University and Shandong Pengbo Biotech Limited Company.

## Conflict of interest

QW and WZ are employed by Shandong Pengbo Biotech Limited Company and Corteva Agriscience, respectively.

The remaining authors declare that the research was conducted in the absence of any commercial or financial relationships that could be construed as a potential conflict of interest.

## Publisher’s note

All claims expressed in this article are solely those of the authors and do not necessarily represent those of their affiliated organizations, or those of the publisher, the editors and the reviewers. Any product that may be evaluated in this article, or claim that may be made by its manufacturer, is not guaranteed or endorsed by the publisher.
