# Supplementary material for: Endophytic extract Zhinengcong alleviates heat stress-induced reproductive defect in Solanum lycopersicum
Source: Front Plant Sci. 2022 Aug 25;13:977881. doi: 10.3389/fpls.2022.977881 (PMC9454194; doi:10.3389/fpls.2022.977881)
Supplement: Supplementary file 1 [file Data_Sheet_1.PDF]

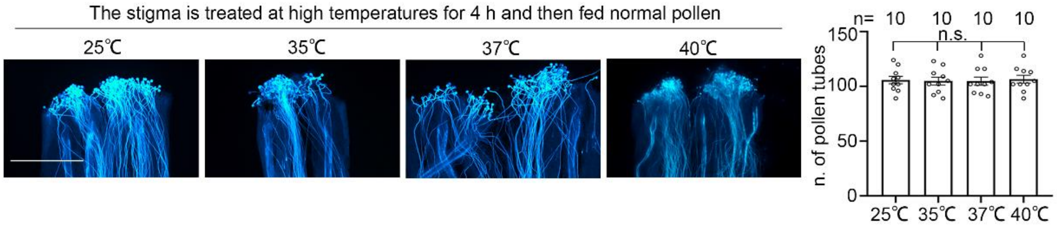

Supplemental Fig. 1 The effect of heat stress on pistils. Scale bar, 500 μm. Bars represent means ± SE m. Asterisk on the top of data bar indicates significant difference (two-tailed t test, *p<0.05, **p<0.01) compared with the data bar on the far left, while asterisk above the bracket represents comparison between the two data bars indicated. The symbol n.s. indicates no difference. All experiments have 3 biological replicates and they have similar results.

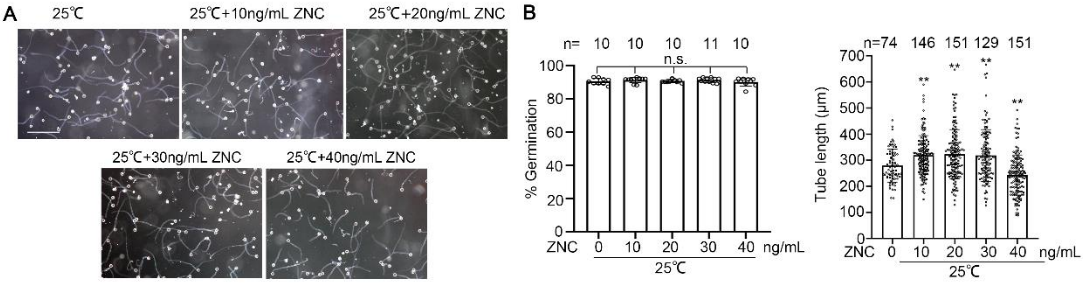

Supplemental Fig. 2 ZNC promotes pollen tube growth at 25 °C. (For Fig. 2)

(A) Pollen growth at 25 °C under ZNC treatment. Scale bar, 200 μm.

(B) Quantification of pollen germination rate and pollen tube length after ZNC treatment at 25 °C. n denotes the number of observation views (left) and pollen tubes (right).

Bars represent means ± SE m. Asterisk on the top of data bar indicates significant difference (two-tailed t test, *p<0.05, **p<0.01) compared with the data bar on the far left, while asterisk above the bracket represents comparison between the two data bars indicated. The symbol n.s. indicates no difference. All experiments have 3 biological replicates and they have similar results.

Supplemental Table 1. The primers used for qRT-PCR

Primer	Sequence
actin-F	5′-TGGTCGGAATGGGA CAGAAG-3′
actin-R	5′-CTCAGTCAGGAGAAC AGGGT-3′
CAT1-F	5′-TGATCGCGAGAAGA TACCTG-3′
CAT1-R	5′-CTTCCACGTTCATGGA CAAC-3′
DHAR-F	5′-CCCTGATGTCCTTG GAGACT-3′
DHAR-R	5′-AAGAACCATTTGGGC TTGTC-3′
Fe-SOD-F	5′-TAAATAGAGACTTT GGTTCC-3′
Fe-SOD-R	5′-TATATTTGCCTCTTAA CCCT-3′
SlRBOHB-F	5′-CAGTGCCCAACAATATCCTC-3′
SlRBOHB-R	5′-TCCTGGTGCTGATGTTATAG-3′
SlRBOHE-F	5′-GAGGAATGAATGTACCACTACAC-3′
SlRBOHE-R	5′-GAGATCCCAAAGGACAAATA-3′
RBOH1-F	5′-TCCAGCACAAGATTACCG-3′
RBOH1-R	5′-CCTCCATTGCGACGAT-3′
SlPIF4-F	5′-CAATCAATCTACAGCGACCA-3′
SlPIF4-R	5′-AGATTTTTGGGCTGACTTGT-3′
